# Hydrogen-Rich Water Treatment of Fresh-Cut Kiwifruit with Slightly Acidic Electrolytic Water: Influence on Antioxidant Metabolism and Cell Wall Stability

**DOI:** 10.3390/foods12020426

**Published:** 2023-01-16

**Authors:** Yanan Sun, Weiyu Qiu, Xiaoqi Fang, Xiaomei Zhao, Xingfeng Xu, Wenxiang Li

**Affiliations:** 1 School of Food Science and Engineering, Qingdao Agricultural University, Qingdao 266109, China; 2 Qingdao Special Food Research Institute, Qingdao 266109, China

**Keywords:** HRW, SAEW, fresh-cut, kiwifruit, antioxidant metabolism

## Abstract

The synergistic impact of hydrogen-rich water (HRW, 394 ppb) and slightly acidic electrolyzed water (SAEW, pH of 6.25 ± 0.19) on the antioxidant metabolism of fresh-cut kiwifruit during storage was investigated (temperature: (3 ± 1) °C, humidity: 80%–85%). Compared with control group, H+S treatment increased the contents of active oxygen-scavenging enzymes (SOD, CAT, POD, and APX) and inhibited the increase of O_2_^•−^ and H_2_O_2_ contents during the storage of fresh-cut kiwifruit. Meanwhile, H+S treatment could reduce the activities of the cell wall-degrading enzymes PG, PME, PL, Cx, and β-Gal, inhibit the formation of soluble pectin, delay the degradation rate of propectin, cellulose, and pseudocellulose, and maintain higher fruit hardness and chewability. The results showed that H+S treatment could enhance free radical scavenging ability and reduce the cell wall metabolism of fresh-cut kiwifruit, maintaining the good texture found in fresh-cut fruit.

## 1. Introduction

Kiwifruit (*Actinidia chinensis*) is not only rich in vitamin C but also contains nutrients needed by the human body, such as dietary fiber, potassium, vitamin E, and folic acid, as well as antioxidants, phytonutrients, and a variety of biologically active ingredients that offer great benefits to health. The vitamin C content in kiwifruit is higher than that in other fruits; one study found that the vitamin C content in Sunshine Golden kiwifruit was almost three times that found in oranges and strawberries [1], which can improve the bioavailability of micronutrient iron. Kiwifruit has a higher antioxidant capacity than apples, grapefruit, and pears, and regular consumption of kiwifruit can help reduce the risk of cardiovascular disease. Compared with whole fruits, fresh-cut fruits and vegetables are more favored by consumers because of their convenience, freshness, and lack of waste. However, it is easy to cause cutting damage to kiwifruit in the process of fresh cutting, such as tissue softening, browning, and loss of nutrients, seriously shortening its shelf life [2,3]. In addition, due to the high water content, juice leakage from fresh-cut kiwifruit would stimulate microbial growth, which may lead to foodborne hazards, seriously hindering the successful development of the industry.

Hydrogen-rich water (HRW) has received increasing attention in the field of fresh food processing and preservation. In animals, H_2_ plays a beneficial role in many physiological and pathological processes, including antioxidant activity and anti-apoptotic protection. In plants, H_2_ can be used as a bioactive gas molecule that promotes seed germination. Chen et al. [4] treated paddy and mung bean with HRW and found that low HRW concentrations could improve the seed germination rate, while high HRW concentrations inhibited germination [4]. The HRW concentrations required for affecting the germination rate of different kinds of seeds are different. H_2_ is also considered to be an anti-stress molecule that is capable of alleviating various abiotic stresses by regulating the antioxidant defense system. For example, Guan et al. [5] found that HRW treatment could improve the germination rate of black barley at lower temperatures (10–15 °C), improve the bioactive ingredients content in black barley germination (including free vanillic acid, conjugate coumaric acid, sinapic acid, and vanillic acid), and enhance the in vitro antioxidant activity [5]. In recent years, the application of H_2_ in fruit and vegetable preservation has attracted widespread attention due to its strong antioxidant activity and permeability. Chen et al. [6] explored the theory that 25% of HRW pretreatment could effectively inhibit the rot of *Eusculus eusculus*, maintain high hardness, improve the activity of antioxidant enzymes, and maintain freshness [6]. High permeability and residue-free properties make H_2_ popular; more importantly, H_2_ is a non-toxic gas compared to NO and H_2_S and does not react with most compounds at room temperature.

Electrolytic water (EW) can be divided into strongly acidic electrolytic water (AEW, pH 2.4–2.9, ACC 31–201 mg/L) and slightly acidic electrolyzed water (SAEW, pH 5.1–6.6, ACC 11–31 mg/L) according to the differences in pH and effective chlorine concentration (ACC) [7]. In recent years, compared with AEW, SAEW has attracted much attention due to its strong antibacterial activity and lack of adverse effects on human health and the environment [8,9]. Today, SAEW is used as a disinfectant for food safety and in preservation [10]. The effective form of the chlorine compounds in SAEW is hypochlorous acid (HClO), which has strong bacteriostatic activity. Issa-Zacharia et al. [11] confirmed that the bacteriostatic activity of SAEW is mainly caused by the potential oxidative damage of HClO to biomolecules [12]. Compared with acid preservatives, SAEW could reduce the environmental damage and corrosive effects of the food industry [13].

Due to the extremely unstable chemical properties of SAEW, exposure to air and light may affect the ability of SAEW to kill microorganisms, seriously affecting its timeliness [14,15]. However, when SAEW is combined with other technologies, this may have additional influences on the bactericidal effect. At present, the stability and feasibility of SAEW when combined with ascorbic acid [16] and plasma water [17] have been reported. There are many studies on the application of SAEW in aquatic products, most of which focus on the reduction of pathogenic microorganisms, but there are few studies on the effect of SAEW treatment on the storage quality of fresh-cut fruits. Therefore, in this paper, the effects of HRW and SAEW on reactive oxygen species (ROS) metabolism and the cell-wall stability of fresh-cut kiwifruit were studied, and the mechanism of HRW and SAEW in fresh-cut kiwifruit preservation was discussed, providing a theoretical basis for maintaining fresh-cut kiwifruit preservation quality.

## 2. Materials and Methods

### 2.1. Preparation of SAEW and HRW

SAEW (pH 6.25, ACC 30 mg/L) was obtained by electrolysis of a 10% hydrochloric acid solution prepared using aqueous electrolysis equipment, with the parameters controlled using pH measurement equipment (PHS-3C, SH Leici, Shanghai, China) and an effective chlorine detector (RC-3F, KRK Kasahara, Kuki, Japan). HRW with an H_2_ concentration of 394 ppb was obtained via electrolysis in a hydrogen-rich water cup.

### 2.2. Materials and Processing

Kiwifruits were purchased from Qingdao Haofeng Food Group Co., Ltd. (Jiaozhou, China); they were selected for their uniform size, greenness, and freedom from diseases and pests. The whole fruits were washed, dried, and peeled, then cut into 6–8 mm slices (about 10–15 g) with a stainless-steel knife. All the slices were randomly divided into 4 groups, each group being 1000 g, and were treated as follows: (1) soaking in sterile distilled water for 5 min (CK); (2) soaking in SAEW for 5 min (SAEW); (3) soaking in HRW for 5 min (HRW); (4) soaking for 5 min in SAEW and HRW, mixed at 1:1 (H+S). After the above four groups of treatment, the processed fresh-cut kiwifruit slices were taken out, placed on the shelf to remove moisture, then stored in a 17.5 × 13.5 × 7.5 cm crisper, under the conditions of 4 ± 1 °C storage. After 0 d, 2 d, 4 d, 6 d, 8 d, and 10 d of storage, slices from each group were randomly sampled to determine the indexes.

### 2.3. Quantification of ROS Antioxidant Metabolism

#### 2.3.1. Determination of O_2_^•−^ and H_2_O_2_ Contents

The determination of H_2_O_2_ content was modified according to Cheng et al. [18]. The pre-cooled acetone was added to the 2 g sample, then ground and centrifuged to obtain the supernatant. First, 1 mL of supernatant, 0.1 mL of TiCl_4_ solution (10%), and 0.2 mL of concentrated ammonia water were shaken several times, centrifuged, and then left to precipitate. Then, 3 mL of 2 mol/L concentrated H_2_SO_4_ was added to dissolve the precipitation, and the supernatant was obtained via centrifugation. By measuring the absorbance value of the supernatant at a wavelength of 412 nm, the content of H_2_O_2_ in the sample was calculated; the results are expressed in μmol/g.

#### 2.3.2. Determination of SOD, CAT, POD, APX Activities

A superoxide dismutase (SOD) assay kit, catalase (CAT) assay kit, peroxidase (POD) assay kit, and ascorbate peroxidase (APX) assay kit were obtained from the Nanjing Jiancheng Institute of Biological Engineering, China, for measuring enzyme activity.

### 2.4. Cell Wall Antioxidant Metabolism

#### 2.4.1. Texture Analysis

A cylindrical (P/75) probe with a diameter of 75 mm was used for the texture analysis (TPA) (TAXT2i, Stable Micro Systems Ltd., London, UK) in accordance with the procedure followed by Cheng et al. [18]. The test conditions were as follows: the pre-speed was 5.0 mm/s, the test speed was 2.0 mm/s, the post-speed was 1.0 mm/s, the penetration distance was 3 mm, the interval between the two cycles was 5 s, and the trigger force was 1.0 N.

#### 2.4.2. Determination of Pectin, Cellulose, and Hemicellulose Contents

The carbazole colorimetric method was adopted, in accordance with the study by Lohani et al. [19], slightly modified for the determination of pectin; the results were expressed in mg/g. The content of hemicellulose in the fruit cell wall was determined by the anthrone colorimetric method, and the result was in mg/g. The content of cellulose in the fruit cell wall was determined by a weighing method, and the result was expressed as a percentage.

#### 2.4.3. Determination of Activity of Cell Wall-Degrading Enzymes (PG, PME, PL, Cx, and β-Gal)

For the enzyme solution preparation, 12 mL of buffer solution was added to 5 g of sample, ground, and homogenized at 4 °C, then centrifuged at 12,000× *g* at 4 °C for 30 min, and, finally, the supernatant was retained.

PG activity and Cx activity were determined by the DNS colorimetric method; the result was expressed as mg/(g·h). PME activity was determined via NaOH titration, and the results were expressed as μg/(g·min).

PL activity determination: After 2 mL of 0.5% pectin solution was heated at 40 °C for 5 min, 0.5 mL of enzyme extract was added and held at 40 °C for 10 min. The mixture of 0.5 mL and 4.5 mL 0.01 mol/L of HCl was mixed and shaken. The absorbance value of the mixture was measured at 235 nm, then the result was expressed as OD235/(g·min).

β-Gal activity determination: 1.5 mL of 1% salicin solution and 0.5 mL of enzyme extract were shaken several times and then thoroughly mixed. After the mixture was fully reacted at 37 °C, 1.5 mL of DNS was quickly incorporated into the sample mixture and then it was boiled in a water bath for 5 min. After that, the cuvette was rinsed with cold water to achieve rapid cooling; the mixture absorbance was measured at 540 nm and the outcome was expressed as μg/(g·min).

### 2.5. Statistics

A one-way analysis of variance (ANOVA) was performed on the experimental data. Median and normal deviations were reported. The differences between means were tested using Duncan’s new multiple range test, with a confidence level of 95%. SPSS 20 (CoStat, Version 6.451, CoHort Software, Pacific Grove, CA, USA) was utilized in the calculation of all statistics. All experiments were performed in triplicate unless otherwise specified.

## 3. Results

### 3.1. Changes in the O_2_^•−^ and H_2_O_2_ Contents of Fresh-Cut Kiwifruit by Different Treatments

**Figure 1:**
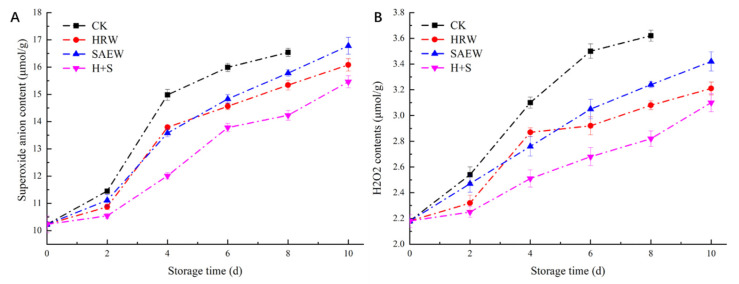
Changes in superoxide anion content (**A**) and H_2_O_2_ content (**B**) in fresh-cut kiwifruit after different treatments, during storage.

The influence of different treatments on the O_2_^•−^ content of fresh-cut kiwifruit during storage is shown in Figure 1A. The O_2_^•−^ content of fresh-cut kiwifruit in each treatment group tended to increase as the storage period was extended. The O_2_^•−^ content of samples processed with HRW, SAEW, and H+S were lower than CK, and the O_2_^•−^ content of fresh-cut kiwifruit treated with H+S was lower than that of HRW and SAEW alone. On the 8th day, the O_2_^•−^ content of fresh-cut kiwifruit treated with HRW, SAEW, and H+S was 92.7%, 95.4%, and 86.0% of CK, respectively. This showed that all three treatments could inhibit the increase in the O_2_^•−^ content of fresh-cut kiwi fruit, and the H+S treatment had the most significant effect (*p* < 0.05).

The consequences of the different disposal methods on the H_2_O_2_ content of fresh-cut kiwifruit during storage is shown in Figure 1B. It can be seen that the H_2_O_2_ content of fresh-cut kiwifruit continued rising with storage time in each treatment group. On the 8th day of storage, the H_2_O_2_ content of fresh-cut kiwifruit treated with HRW, SAEW, and H+S were 85.1%, 89.5%, and 77.9% of CK, respectively. This result showed that the H+S treatment could significantly inhibit the increase in H_2_O_2_ content (*p* < 0.05) and reduce its damage to fresh-cut fruit.

### 3.2. Changes in SOD, CAT, POD, and APX Activities after Different Treatments

**Figure 2:**
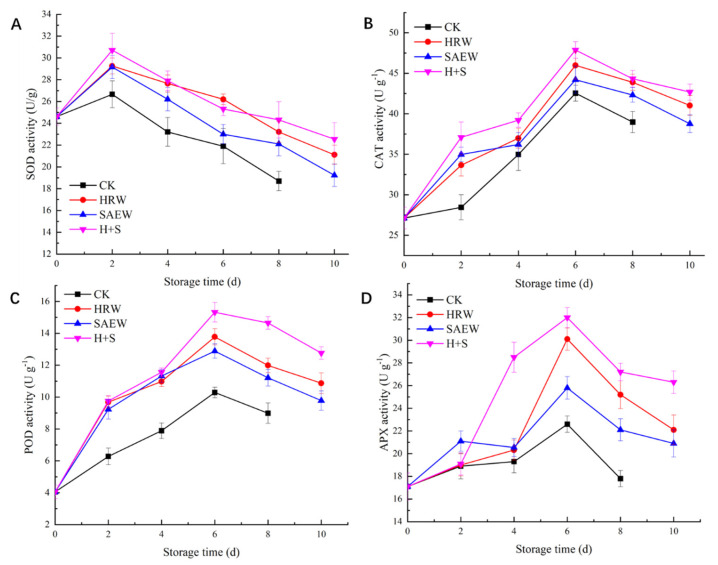
SOD activity (**A**), CAT activity (**B**), POD activity (**C**), and the APX activity (**D**) of fresh-cut kiwifruit after different treatments during storage.

The impact of various treatments on the SOD activity of fresh-cut kiwifruit during storage is shown in Figure 2A. Throughout the storage period, the SOD activity of the samples in each treatment group reached a peak value on day 2 and then gradually decreased. The SOD activity of fresh-cut kiwifruit treated with HRW, SAEW, and H+S was significantly higher (*p* < 0.05) than that of CK during storage. The SOD activity of samples treated with H+S was higher than that of samples treated with HRW and SAEW, except for the 6th day. The SOD activity of fruits treated with HRW, SAEW, and H+S was 1.24, 1.18, and 1.30 times higher than that of CK, respectively, after storage for the 8th day. The above analysis showed that the H+S treatment can effectively improve the SOD activity and delay the decay of fresh-cut kiwifruit.

The change in the CAT activity of fresh-cut kiwifruit during storage is shown in Figure 2B. During storage, the CAT activity of the sample in each group gradually increased in the early stages and began to decline gradually after reaching the maximum value on the 6th day. During the storage, the CAT activity of the CK group of fresh-cut kiwifruit was always lower than that of other treatment groups, and the HRW, SAEW, and H+S treatments significantly increased the CAT activity of fresh-cut kiwifruit (*p* < 0.05). On the 6th day, the CAT activity of the samples treated with HRW, SAEW, and H+S was 45.98 U/g, 44.21 U/g, and 47.87 U/g, respectively, which was significantly higher than that of the CK group (*p* < 0.05). It indicates that the CAT activity of fresh-cut kiwifruit was effectively improved after HRW, SEW, and H+S treatment, and the H+S treatment was the best.

As shown in Figure 2C, throughout the storage process, the POD activity of fresh-cut kiwifruit in each group increased rapidly in the early storage period and reached a peak on the 6th day. Throughout the storage process, the POD activity of the samples in the CK group was significantly less than those in the other treated groups (*p* < 0.05). The POD activity of fresh-cut kiwifruit treated with HRW, SAEW, and H+S showed no significant difference during the first six days of storage (*p* > 0.05). From the 6th day, the effect of H+S treatment on the POD activity of the sample was significantly higher than that of the HRW and SAEW treatment groups (*p* < 0.05). On the 8th day of storage, the POD activity of fresh-cut kiwifruit treated with HRW, SAEW, and H+S were 1.33 times, 1.25 times, and 1.63 times that of the CK group samples, respectively.

As seen in Figure 2D, the APX activity of fresh-cut kiwifruit first increased internally and then decreased with storage time, reaching a peak value at day 6. During the storage, except for the second day, the APX activity of samples treated with HRW, SAEW, and H+S was significantly higher than that of the CK group sample (*p* < 0.05). Since then, the APX activity of samples treated with H+S has been at a high level. Until the 6th day of storage, the APX activity of samples treated with HRW, SAEW, and H+S was 1.33 times, 1.17 times, and 1.42 times that of the CK group samples, respectively. The APX activity of fresh-cut kiwifruit treated with H+S was significantly increased (*p* < 0.05), which is beneficial for maintaining fruit quality.

### 3.3. Changes in the Hardness, Elasticity, and Chewability

The firmness, elasticity, and chewiness of fresh-cut kiwifruits showed a declining trend during storage (Table 1), which was due to fruit softening caused by tissue aging. It can be seen from the table that the firmness and chewiness of the fruit decreased significantly after 8 days of storage, while the elasticity of all samples remained stable. The sample hardness of the H+S treatment group was always at a higher level compared with other treatment groups, and the fruit hardness of the H+S treatment group was 5.1 N, which was 2.2 times that of the CK treatment group when it was stored for 8 days. The elasticity value remained stable during storage and fluctuated slightly between the different treatment groups, but there was no significant difference. The chewiness value reflected the anti-masticatory property of the fruit. The results showed that the masticatory property of the fruit in the CK group decreased significantly during storage. The SAEW, HRW, and H+S treatments could significantly delay (*p* < 0.05) the decline in the masticatory properties to varying degrees, among which the H+S treatment had the best effect.

### 3.4. Changes in the Contents of Pectin, Soluble Pectin, Cellulose and Hemicellulose

**Figure 3:**
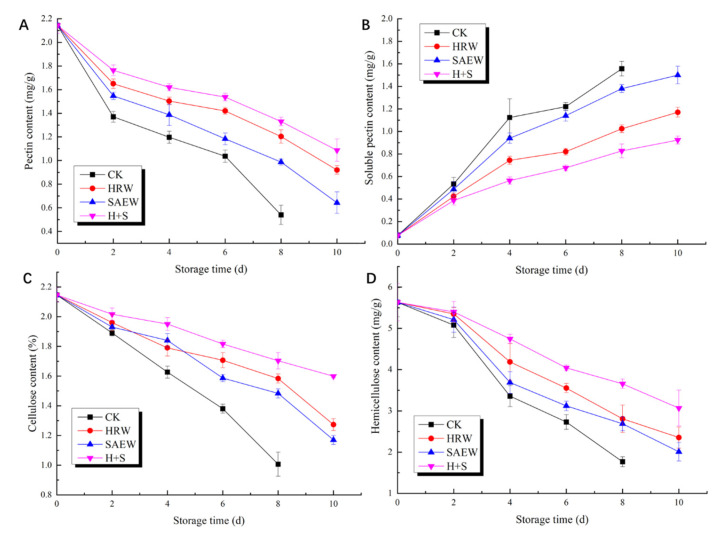
Pectin content (**A**), soluble pectin content (**B**), cellulose content (**C**), and hemicellulose content (**D**) of fresh-cut kiwifruit, with different treatments during storage.

The influence of the different processes on the content of pectin in fresh-cut kiwifruit is shown in Figure 3A. During storage, the content of the pectin in the sample was gradually reduced, due to the continuous decomposition. HRW, SAEW, and H+S treatments could delay the decline in the pectin content of fresh-cut kiwifruit, to varying degrees. The pectin content of the H+S treatment sample was always at a high level, and the pectin content of fresh-cut kiwifruit after the H+S treatment was 1.33 mg/g after storage until the 8th day, which figure was 2.46 times higher than that in the CK group.

As indicated in Figure 3B, the changing trend of the soluble pectin content of fresh-cut kiwifruit was opposite to that of the protopectin content, and the soluble pectin content increased continuously during storage. Compared with the CK samples, HRW, SAEW, and H+S treatments could significantly (*p* < 0.05) delay the increase in the soluble pectin content in fresh-cut kiwifruit. Until the 8th day, the soluble pectin content in fresh-cut kiwifruit treated with HRW, SAEW, and H+S were 65.4%, 88.5%, and 53.2% of the CK samples, respectively. In all these ways, H+S treatment demonstrated the best effect, and the pectin content in the fruit was only half that of the CK samples.

It can be seen from Figure 3C that the cellulose content of fresh-cut kiwifruit was constantly decreasing during the storage period. At the early stage of storage, there was no significant difference in the cellulose content of fruit among the treatments of each group (*p* > 0.05). With the extension of storage time, the cellulose content of the sample under the H+S treatment was at a higher level. By the 8th day of storage, the cellulose content of fresh-cut kiwifruit under HRW, SAEW, and H+S treatments was 1.58 times, 1.48 times, and 1.70 times higher than that under CK treatment, respectively.

The hemicellulose content of fresh-cut kiwifruit decreased continuously during the whole storage period (Figure 3D). HRW, SAEW, and H+S treatments can delay the decrease in fruit hemicellulose to varying degrees, among which H+S treatment has the best effect. On the 8th day after storage, the cellulose content of fresh-cut kiwifruit treated with HRW, SAEW, and H+S was 1.59 times, 1.52 times, and 2.07 times higher than that of CK, respectively.

### 3.5. Effects of Different Treatments on PG, PME, PL, and Cx and β-Gal Activity

**Figure 4:**
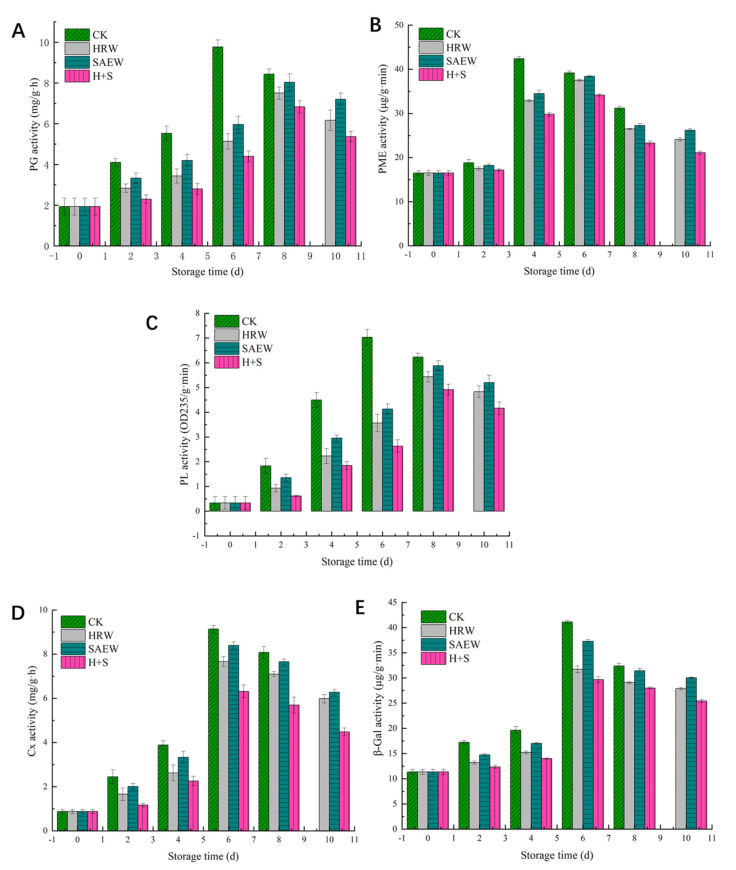
PG activity (**A**), PEM activity (**B**), PL activity (**C**), Cx activity (**D**), and β-Gal activity (**E**) of fresh-cut kiwifruit given different treatments during storage.

Fresh-cut kiwifruit PG activity under different treatments is shown in Figure 4A. The PG activity of the fruit first increased throughout the storage period and then decreased with the increasing storage time. However, the PG activity of the sample in HRW, SAEW, and H+S treatments was below that of the CK group throughout the storage period. The PG activity of fresh-cut kiwifruit in the CK group reached its peak on the 6th day, being 1.9 times, 1.64 times, and 2.22 times higher than that in the HRW, SAEW, and H+S treatments, respectively. The H+S treatment samples reached a peak on the 8th day, which was 2 days later than in the CK group, which showed that H+S treatments could significantly reduce the PG activity of fresh-cut kiwifruit (*p* < 0.05).

It is evident from Figure 4B that the PME activity of fresh-cut kiwifruit first increased and then decreased with increasing storage time. After 2 days of storage, the PME activity of fruit treated with HRW, SAEW, and H+S were 17.53 μg/g·min, 18.23 μg/g·min, and 17.17 μg/g min, respectively. No significant differences were found between them (*p* > 0.05). The PME activity of fruit in the CK treatment reached its maximum value on day 4, compared with other treatment groups, The H+S treatment significantly suppressed the increase in PME activity (*p* < 0.05), and the fruit PME activity of the H+S treatment group on day 4 was only 70.3% of that of the CK group. These results suggest that H+S treatment could delay the degradation of pectin substances by reducing PME activity in fresh-cut kiwifruit.

Figure 4C shows the effect of different treatments on the PL activity of fresh-cut kiwifruit. The PL activity of the samples increased and then decreased as the storage time increased, and the PL activity of the samples treated with HRW, SAEW, and H+S was lower than that of the samples treated with CK throughout the storage period. The PL activity of the fruits in the CK group peaked on day 6, while that of the other three treated samples peaked on day 8. The peak PL activity of fresh-cut kiwifruit treated with CK, HRW, SAEW, and H+S was 7.03 OD235/g·min, 5.43 OD235/g·min, 5.88 OD235/g·min and 4.92 OD235/g·min, respectively.

The influence of different treatments on the Cx activity of fresh-cut kiwifruit is shown in Figure 4D. During storage, the Cx activity of the fruit first increased and then decreased, reaching its peak value on the 6th day. The Cx activity of the sample in the CK group was significantly higher than that in other treatment groups (*p* < 0.05), which was 1.19 times, 1.09 times, and 1.45 times higher than that in the HRW, SAEW, and H+S treatment groups, respectively, at the 6th day of storage.

Fresh-cut kiwifruit β-Gal activity first increased and then decreased with the prolongation of storage time, as shown in Figure 4E. The sample β-Gal activity when treated with HRW, SAEW, and H+S was lower than in the CK group, and in fruit treated with H+S, the β-Gal activity was higher than that of the other two treatments during storage. Fresh-cut kiwifruits in each group were stored until the 6th day, when β-Gal activity reached its peak, and the fruit was treated with HRW and SAEW. H+S β-Gal activity was 77.2%, 90.66%, and 72.1% that of the CK group, respectively.

## 4. Discussion

The ripening and senescence of fruits and vegetables are closely related to ROS; the production rate and clearance rate of ROS in organisms can reach a relative balance under undamaged conditions. However, in the senescence of fruits and vegetables, the clearance rate of free radicals could be lower than the production rate of free radicals in the body. At this time, the balance would be broken, which leads to the accumulation of ROS in fruit [9]. The main reason for the ripening and senescence of fruits and vegetables is that an increase in ROS, especially the accumulation of O_2_^•−^ and H_2_O_2_, causes oxidative damage to the biomolecules, such as lipid peroxidation, and will ultimately lead to cell death [20]. This study found that the contents of O_2_^•−^ and H_2_O_2_ in fresh-cut kiwifruit increased rapidly in the early stages of storage (Figure 1), which is related to the decreasing function of the ROS scavenging system in the fruit. Compared with the CK group, HRW and SAEW have a positive effect on ROS removal and should keep them at a low level; the H+S treatment could reduce the content of O_2_^•−^ and H_2_O_2_ in fresh-cut kiwifruit to a large extent, alleviating the oxidative stress of fruit.

Enhancing the activity of the ROS-scavenging enzymes (SOD, CAT, POD, and APX) is a common mechanism for eliminating ROS in fruit cells. SOD plays an important role in the ROS clearance of fruit and vegetable tissue, which is the first line of defense for eliminating ROS. It catalyzes the disproportionate nature of O_2_^•−^ into H_2_O_2_ and molecular oxygen, and it is the only enzyme for O_2_^•−^ scavenging [21]. The CAT, POD and APX enzymes work together to decompose H2O_2_ into H_2_O and O_2_ in fruits and vegetables [22]. In this study, H+S treatment significantly improved the SOD, CAT, POD, and APX activities of fresh-cut kiwifruit (Figure 2), indicating that the antioxidant activity was enhanced. One of the functions of HRW is probably that H_2_ can easily penetrate the cell membrane, thus increasing the antioxidant gene expression of the encoding SOD, CAT, POD, and APX [23,24]. The effect of SAEW may be that the chloride in SAEW stimulates the oxidation of fruit [25].

The firmness of the fruit decreases continuously during storage, which is closely related to structural changes in the cell wall, mainly due to the hydrolysis of the pectin polymer in the pulp cell wall. This softening of the fruit is caused by an increase in the cell-wall metabolizing enzymes (PME, PG, PL, Cx, and β-Gal), resulting in the gradual degradation of cell-wall material components [26,27]. The fresh-cut fruit’s firmness decreased continuously during storage; the H+S treatment group sample showed the highest firmness and chewiness value (Table 1), while the elasticity value remained stable during storage. At the early stages of storage, fresh-cut kiwifruit had a high hardness, and its cell wall was mainly composed of protopectin, hemicellulose, and cellulose. With the increase in storage time, the fruit appeared post-ripening and softening, and the protopectin in the fruit was continuously degraded (Figure 3), while the soluble pectin was constantly increasing in this process. In addition, the content of cellulose and hemicellulose also continued to decline, which is consistent with the research results of Langer et al. [28].

The main reason for the texture-softening of fruit is that the cell wall-degrading enzyme acts on the cell wall, reducing the connectivity degree between cells, and resulting in cell separation [29]. PG and PME are closely related to the decomposition of pectin in the fruit cell wall. The acting substrate of PG is the polygalacturonic acid in the cell wall, which is hydrolyzed into galacturonic acid or its oligomers. PME acts on the carboxyl group of pectin molecules to convert it into polygalacturonic acid, which is used as the hydrolysis substrate of PG. Peak PME activity appeared on day 4 for control samples and on day 6 for the HRW, SAEW, and H+S treatment groups, while the peak PG activity appeared on day 6 for the control group and on day 8 for the other treatment groups, indicating that the peak PME activity appeared earlier than PG, which is consistent with the results of previous studies [30,31].

## 5. Conclusions

(1) Compared with the CK group, the H+S treatment significantly (*p* < 0.05) reduced the content of free radicals (O_2_^•−^ and H_2_O_2_) in fresh-cut kiwifruit during storage, increased the activities of the ROS scavenging enzymes, SOD, CAT, POD, and APX, and improved the antioxidant capacity of fruit.
(2) During storage, the firmness and chewability of fresh-cut kiwifruit decreased continuously, and the protopectin and cellulose were degraded. Compared with the CK group, the H+S treatment significantly increased (*p* < 0.05) the content of protopectin, cellulose, and hemicellulose in fresh-cut kiwifruit during storage, inhibited the increased rate of soluble pectin, delayed the decomposition of cell-wall substances, and maintained the integrity of the fruit cell wall.
(3) The decline in fruit firmness is related to the activity of cell wall-degrading enzymes. The H+S treatment reduced the PG, PME, PL, Cx, and β-Gal enzyme activity, and inhibited the degradation of pectin, cellulose, and hemicellulose, which are the main components of the cell wall so that the softening of fresh-cut kiwifruit tissues was delayed.

## Figures and Tables

**Table 1:** Changes in the hardness, elasticity, and chewiness of fresh-cut kiwifruit during storage after different treatments.

	Storage Time (d)	H+S	HRW	SAEW	Control
Hardness (N)	0	9.21 ± 0.28 ^a^	9.21 ± 0.28 ^a^	9.21 ± 0.28 ^a^	9.21 ± 0.28 ^a^
	2	8.21 ± 0.32 ^d^	7.94 ± 0.25 ^c^	7.02 ± 0.31 ^b^	6.31 ± 0.15 ^a^
	4	7.72 ± 0.24 ^d^	7.14 ± 0.15 ^c^	6.20 ± 0.13 ^b^	5.67 ± 0.15 ^a^
	6	6.91 ± 0.18 ^c^	6.10 ± 0.47 ^b^	4.54 ± 0.28 ^a^	3.98 ± 0.10 ^a^
	8	5.12 ± 0.19 ^c^	4.12 ± 0.32 ^b^	2.65 ± 0.11 ^a^	2.32 ± 0.12 ^a^
	10	3.89 ± 0.22 ^c^	3.32 ± 0.16 ^b^	2.33 ± 0.17 ^a^	
Elasticity	0	0.7 ± 0.03 ^a^	0.7 ± 0.03 ^a^	0.7 ± 0.03 ^a^	0.7 ± 0.03 ^a^
	2	0.69 ± 0.07 ^a^	0.65 ± 0.05 ^a^	0.66 ± 0.03 ^a^	0.66 ± 0.06 ^a^
	4	0.67 ± 0.01 ^a^	0.66 ± 0.09 ^a^	0.67 ± 0.02 ^a^	0.66 ± 0.03 ^a^
	6	0.65 ± 0.07 ^a^	0.64 ± 0.01 ^a^	0.64 ± 0.04 ^a^	0.62 ± 0.02 ^a^
	8	0.64 ± 0.03 ^a^	0.62 ± 0.03 ^a^	0.63 ± 0.02 ^a^	0.61 ± 0.01 ^a^
	10				
Chewiness (N)	0	9.78 ± 0.55 ^a^	9.78 ± 0.55 ^a^	9.78 ± 0.55 ^a^	9.78 ± 0.55 ^a^
	2	9.31 ± 0.56 ^c^	8.99 ± 0.56 ^b^	8.43 ± 0.38 ^a^	8.12 ± 0.47 ^a^
	4	7.89 ± 0.47 ^c^	7.46 ± 0.61 ^b^	7.20 ± 0.31 ^b^	6.21 ± 0.44 ^a^
	6	7.46 ± 0.39 ^d^	6.99 ± 0.22 ^c^	6.41 ± 0.34 ^b^	5.89 ± 0.36 ^a^
	8	7.21 ± 0.33 ^c^	6.45 ± 0.38 ^b^	5.58 ± 0.51 ^a^	5.21 ± 0.37 ^a^
	10	6.89 ± 0.24 ^b^	5.69 ± 0.25 ^a^	5.45 ± 0.22 ^a^	

^a–d^ Different letters, marked on values of the same sampling time, indicate significant differences between treatments (*p* < 0.05).

## Data Availability

Data is contained within the article.
